# Dual-task performance in old adults: cognitive, functional, psychosocial and socio-demographic variables

**DOI:** 10.1007/s40520-021-02002-x

**Published:** 2021-10-14

**Authors:** María Campos-Magdaleno, Arturo Pereiro, Esperanza Navarro-Pardo, Onésimo Juncos-Rabadán, David Facal

**Affiliations:** 1 Department of Developmental Psychology, University of Santiago de Compostela, Santiago, Spain; 2 Department of Developmental Psychology, University of Valencia, Valencia, Spain; 3 Facultade de Psicoloxía, Rúa Xosé María Suárez Núñez, s/n. Campus Vida, 15782 Santiago de Compostela, Galicia Spain

**Keywords:** CogniFraSp, Dual task, Fluency, Tracking, Frailty, Community-dwelling old adults

## Abstract

**Background:**

Dual tasking, or the ability to executing two tasks simultaneously, has been used in recent research to predict cognitive impairments, physical frailty, and has been linked with cognitive frailty in old adults.

**Aim:**

This study aimed to determine age-related variables can predict dual-task (DT) performance in the older population.

**Methods:**

A total of 258 healthy community-dwelling participants + 60 years were assessed in relation to their functional capacity, health, well-being, social support and years of education. Performance of a cognitive (Fluency) task and a cognitive–motor (Tracking) task was recorded under single and DT conditions. Multiple linear regression analysis was carried out for each dependent variable, in separate models including cognitive, functional and psychosocial variables.

**Results:**

Performance in Fluency in DT conditions was predicted by cognitive variables, whereas performance in Tracking DT conditions was predicted by positive interaction, health status, age and motor variables.

**Discussion:**

The findings suggest that a wide range of cognitive, psychological, social, physical and functional variables influence cognitive and motor performance in aging.

**Conclusion:**

DT methodology is sensitive to different age-related changes and could be related to frailty conditions in aging.

## Introduction

Dual tasking consists of the simultaneous execution of two tasks, generally cognitive and/or motor tasks of different levels of complexity (for example, maintaining a conversation while walking). In dual-task (DT) studies, the nature of the separate tasks must have distinct goals, and they must be performed and measured separately [1]. The underlying assumption of DT studies is that processing resources are limited and resource competition occurs in cognitive performance in daily life [2]. Under DT conditions, performance of one or both tasks can vary relative to single conditions because of competing demands, due to insufficient attentional resources or an inability to share these limited attentional resources between two or more concurrent tasks, thus causing a decline in performance [3].

The DT methodology has been used for different research purposes, mainly to investigate the executive capacities required to share attentional resources. In gerontology, resource competition has been found to be particularly pronounced in older adults, and the DT paradigm has emerged as a promising tool for predicting cognitive impairment [4] and frailty status [5] in realistic situations. For instance, dual tasking has been used to differentiate between old adults at risk and not at risk of falling [6]. Using a combination of motor (walking) and cognitive (fluency and countdown) tasks, Cadore et al. [7] and Martínez-Ramírez et al. [8] detected mean differences in gait performance between frail and robust participants. Studies using combinations of similar DT conditions (gait as motor task; subtractions, countdown or fluency as cognitive tasks) showed that gait performance differs between people with cognitive impairment and normal cognition [6, 9, 10]. In participants with cognitive impairment or poor health status, greater difficulties were expected in the coordinated allocation of processing resources and, consequently, in performing usual daily-living tasks under DT conditions [9, 11, 12].

DT has been proposed as an appropriate marker for assessing cognitive frailty [6, 13]. Cognitive frailty is an emerging topic in studies of older adults at risk of developing disability and/or comorbidity. The concept of frailty, understood as a physical status characterized by a decreased biological reserve that leads to a major risk of adverse health [14], has also been linked to cognitive and psychosocial variables of aging [15, 16]. Facal and colleagues [17] highlighted the importance of motor performance in the relationship between physical disability and cognitive impairment. In recent years, psychosocial and environmental characteristics have been added as possible factors related to frailty, as part of the concept of accumulation deficits [18–20]. Although social frailty is a seldom studied concept [19], it has been found that social environment might play a role in preventing or reducing health-related adverse outcomes in later life [16]. The observed relationship between lack of support network and increasing health-related risks in aging has been suggested to be due to the preventive role of social participation, or rather that frail people are less likely to participate in social activities [21]. Psychological symptoms are also important factors associated with pathological aging beyond cognitive impairment [20, 22] including negative affect, anxiety, sadness, depressive symptoms [20] and subjective well-being [23, 24]. Educational level is another characteristic that has been shown to play an important role in research, as longer periods of schooling have been associated with possession of greater personal resources to deal with risk situations [12, 20, 25, 26].

The aim of the present study was to determine the cognitive, physical/functional, psychosocial and socio-demographical variables that influence performance of a dual task, composed of a verbal-cognitive (Fluency) task and a cognitive–motor (Tracking) task in community-dwelling old adults without cognitive impairment. Our hypothesis is that DT performance in both tasks will be at least partly influenced by variables generally considered in a broad model of frailty, such as cognitive status, functional capacity, health, well-being, social support, age and years of education.

## Methods

### Participants

A total of 258 healthy community-dwelling participants aged 60 years and over (mean = 70.58, range 60–90, women = 50%) were included in the study. The participants were recruited from two regions of Spain (Galicia in the northwest, and Valencia in the southeast), within the CogniFraSp (Cognitive Frailty Spain) study. Inclusion criteria were as follows: (1) age 60 years old or over; (2) lack of previous diagnosis of dementia and other major mental disease; (3) lack of severe traumatic injury (or consequences) that hinder or impede the assessment; and (4) lack of severe gait disturbance (i.e., elevated risk of falling, inability to walk more than 10 m unaided) and lack of use of external technical aids (sticks, crutches, walkers, wheelchairs, etc.). Participants with a cognitive status below the Mild cognitive impairment (MCI) cut-off considering norms of age and education and measured by Montreal Cognitive Assessment (MoCA [27]; normative data from the Spanish version [28]) were also removed from the sample. The demographic and neuropsychological profiles of the participants are summarized in Table 1.

**Table 1:** Mean scores, standard deviations (in parentheses) and range of values for cognitive, physical/functional, psychosocial and socio-demographic variables of the sample (*n* = 258)

Variable	Descriptive values
MoCA	25.83 (2.57); range: 20–31
Fluency ST	12.25 (5.06); range: 2–30
Tracking ST	88.53 (38.38); range: 12–217
Fluency DT	10.27 (5.09); range: 2–27
Tracking DT	60.64 (34.21); range: 0–190
Fluency DTC	− 14.22 (34.99); range: − 76.92/250
Tracking DTC	− 31.08 (26.73); range: − 100/120
TUG	9 (3); range: 5–32
MOS-ES	34.42 (6.91); range: 13–40
MOS-IS	17.29 (4.12); range: 4–20
MOS-PI	17.49 (3.30); range: 4–20
MOS-AS	13.72 (2.03); range: 6–15
GHQ 1	5.81 (1.98); range: 0–15
GHQ 2	3.56 (3.37); range: 0–12
GHQ 3	3.20 (2.15); range: 0–9
VREM classification	Light activity: 3.9% Moderate activity: 4.3% Active: 9.7% Heavy activity: 82.2%
Gender	Men: 50%; Women: 50%
Age	70.58 (7.61); range: 60–90
Years of education	10.23 (4.57); range: 2–20
Education level	Unschooled: 6.6% Basic: 54.7% Secondary: 22.1% University: 16.7%

Gender, education level and VREM classification are expressed as percentages

*MoCA* Montreal Cognitive Assessment, *ST* Single Task, *DT* Dual Task, *DTC *Dual Task Costs, *TUG* Timed Up and Go, *MOS-ES* MOS questionnaire for social survey-Emotional Support; *MOS-IS* MOS questionnaire for social survey-Instrumental Support, *MOS-PI* MOS questionnaire for social survey-Positive Interaction; *MOS-AS* MOS questionnaire for social survey-Affective Support, *GHQ* General Health Questionnaire 12-items, factors 1, 2 and 3, *VREM* Minnesota Leisure Time Physical Activity Questionnaire

Written informed consent was obtained from all participants prior to their participation in the study, which was conducted in accordance with the previsions of the Declaration of Helsinki, as revised in Seoul 2008. The research plan was approved by the Clinical Research Ethics Committee of the Xunta de Galicia (procedure number 2018/620) and the Commission of Ethics in Experimental Research of the University of Valencia (procedure number H1521026499251). Participants did not receive any economic compensation for taking part in the study.

### Procedure and materials

The study protocol was designed to assess cognitive frailty in a larger on-going research project, including socio-demographic and health issues, physical capacity, cognitive reserve, cognitive functioning and subjective cognitive complaints. The protocol was administered in a single, 90-min (approximately) session. Recruitment of participants was carried out in cooperation with graduate and postgraduate students in Psychology and Pedagogy faculties in the study regions.

We selected various tests for the present study purposes, as summarized below.

A. We used the total score in the MoCA test [27] (for Spanish norms for age and education: [28]) as a measure of cognitive functioning.
B. We used the following tests to measure physical/functional capacity: (1) the Lawton-Brody Instrumental Activities of Daily Living Scale (LB-IADL) Scale [29] (total score, self-reported information); (2) the Minnesota Leisure Time Physical Activity Questionnaire (VREM, [30]; Spanish version: [31]), which provides an estimate of energy expenditure based on self-reported kilocalories expended per week; participants were classified according to four categories of activity: light, moderate, active and heavy activity; (3) the Timed Up and Go Test (TUG) [32], which scores the time in seconds spent in getting up from a chair without armrests, walking 3 m, returning to the chair and sitting down; and (4) the Charlson Comorbidity Index (CCI) [33], total score.
C. We used the following tests to measure psychosocial variables: (1) the 12-item General Health Questionnaire (GHQ-12 [34]; Spanish version: [35]), which assesses self-perception of health and well-being, applied using a Likert scale (0–3) and scored by factors: Factor 1 “Successful Coping”, which assesses the self-perception of solving problems capacity; Factor 2 “Self-esteem”, which assesses positive self-concept and self-esteem; and Factor 3 “Stress”, which assesses anxiety and stress perception [36]. Scores for Factors 1, 2, and 3 were used as measures; and (2) the MOS questionnaire Social Support Survey [37] (Spanish version: [38]), which assesses social support, using a Likert scale (1–5) and is scored according to four areas: Emotional Support (MOS-ES, concerning affect expressions, emphatic comprehension, expression of feelings, counseling and information); Instrumental Support (MOS-IS, provision of material or tangible assistance); Positive Interaction (MOS-PI, possibilities of meeting and have fun with other people); and Affective Support (MOS-AS; real demonstrations of love and affection); scores in each of the four MOS areas were used as variables.
D. We used the following tests to assess DT performance: (1) a 60-s verbal Phonological Fluency task (P, R or M letters counterbalanced for each participant) [39], in which participants were instructed to orally produce as many words as possible within 60 s beginning with the specific test letters, and were informed of inadmissible words (repetitions, proper names or words with different inflection sharing the same root); and (2) a paper-and-pencil circle tracking task [40] which requires the participant to draw a line through circles arranged in a path around a sheet of paper during one minute (the task includes a previous practice trial); the measure considered was the number of circles correctly crossed by the line. Fluency and tracking were first carried out in a single condition, with each performed separately. The participants were then required to perform both tasks simultaneously for one minute, under the DT condition. Participants did not receive additional instructions about prioritizing one task over the other, and they were encouraged to continue if they stopped before the time allowed for the test. The corresponding variables considered were the number of correct words and the number of crossed circles in the DT condition. DT costs were calculated as percentage of change: DTC = [(STP-DTP)/STP] × 100, where STP is the single-task performance, and DTP the dual-task performance [41].

For the variables described in this section, higher scores on TUG, CCI, and GHQ represent poorer performance. For the other variables, higher scores represent higher performance.

### Statistical analysis

The data were analyzed using the SPSS statistical software package, version 26.0 (SPSS, Chicago IL, USA). To determine which variables can predict the level of performance of Fluency and Tracking in DT conditions, multiple linear regression analysis (MLRA) was applied to the total fluency and total tracking scores in the DT condition as dependent variables. We first performed separately a MLRA by entering different sets of independent variables for each of the dependent variables: MoCA and Fluency in a single condition as cognitive variables; VREM, TUG, CCI and LB-IADL as physical/functional variables; MOS factors (MOS-ES, MOS-IS, MOS-PI and MOS-AS) and GHQ factors 1, 2 and 3 as psychosocial variables; and Age and Years of Education as socio-demographic variables. For the Tracking DT performance, we included the MoCA and Tracking in single condition measures as cognitive variables, and the same physical/functional, psychosocial and socio-demographic variables as for Fluency.

We then repeated the MLRAs for DT Fluency and DT Tracking, this time entering all the cognitive, physical/functional, psychosocial and socio-demographic variables found to be significant in the previous MLRAs, as independent variables, to produce a final multiple-regression predictive model.

## Results

Descriptive data of the sample for demographic and neuropsychological variables are summarized in Table 1.

The results of the MLRAs for Fluency in DT as the dependent variable and cognitive, physical/functional, psychosocial and socio-demographic measures as predictors are shown in Table 2. Fluency in the DT condition was significantly predicted by the following: (a) the cognitive measures MoCA and Fluency in the ST condition (Model 1, *R*^2^ adjusted = 0.546, *F* = 155.37, *p* < 0.01); the physical/functional measures TUG and LB-IADL (Model 2, *R*^2^ adjusted = 0.146, *F* = 11.941, *p* < 0.01); (c) the psychosocial measures MOS-IS and GHQ 1 (Model 3, *R*^2^ adjusted = 0.030, *F* = 2.130, *p* < 0.05); and Age and Years of Education (Model 4, *R*^2^ adjusted = 0.220, *F* = 39.084, *p* < 0.01).

**Table 2:** Multiple regression analysis, with performance in Fluency in the Dual Task condition as the dependent variable and cognitive, physical/functional, psychosocial and socio-demographic measures as independent variables

Variable entered in model	*R*^2^	*R*^2^ adjusted	*F*	*β*	*T*
Model 1 (cognitive)	0.549	0.546	155.37**		
MoCA				0.163	3.446**
Fluency ST				0.652	13.773**
Model 2 (physical/functional)	0.159	0.146	11.941**		
VREM				− 0.047	− 0.780
TUG				− 0.349	− 5.774**
CCI				− 0.088	− 1.504
LB-IADL				0.120	2.013*
Model 3 (psychosocial)	.056	.030	2.130*		
MOS-ES				0.161	1.746
MOS-IS				− 0.185	− 2.356*
MOS-PI				0.047	0.412
MOS-AS				− 0.013	− 0.139
GHQ 1				− 0.134	− 2.112*
GHQ 2				0.119	1.110
GHQ 3				− 0.075	− 0.694
Model 4 (socio-demographic)	0.235	0.220	39.084**		
Years Education				0.460	8.285**
Age				− 0.181	− 3.222**

*MoCA* Montreal Cognitive Assessment, *ST* Single Task, *VREM* Short Form Minnesota Leisure Time Physical Activity Questionnaire, *TUG* Timed Up and Go, *CCI* Charlson Comorbidity Index, *LB-IADL* Lawton-Brody Instrumental Activities of Daily Living Scale, *MOS-ES* MOS questionnaire for social survey-Emotional Support, *MOS-IS* MOS questionnaire for social survey-Instrumental Support, *MOS-PI* MOS questionnaire for social survey-Positive Interaction, *MOS-AS* MOS questionnaire for social survey-Affective Support, *GHQ* General Health Questionnaire 12-items, factors 1, 2 and 3

**p < 0.01, *p < 0.05

The results of the MLRAs for Fluency in DT condition including all the cognitive, physical/functional, psychosocial and socio-demographic predictors that were significant in the previous analyses are shown in Table 3. Only the cognitive measures MoCA and Fluency ST were significant predictors (Model 5a: *R*^2^ adjusted = 0.560, *F* = 41.428, *p* < 0.01). We then performed other MLRAs with those significant variables and obtained a final satisfactory model (Model 5b: *R*^2^ adjusted = 0.546, *F* = 155.369, *p* < 0.01) with the previous two significant measures.

**Table 3:** Multiple regression analysis, with performance in Fluency in Dual Task condition as the dependent variable. The statistical significance of the independent variables in Models 1, 2, 3 and 4 (Model 5a) and the statistical significance of the independent variables in Model 5a (Model 5b) are shown

Variable entered in model	*R*^2^	*R*^2^ adjusted	*F*	*Β*	*t*
Model 5a	0.573	0.560	41.428**		
MoCA				0.178	3.557**
Fluency ST				0.584	10.476**
MOS-IS				− 0.060	− 1.431
GHQ 1				− 0.068	− 1.591
TUG				− 0.076	− 1.509
LB-IADL				0.052	1.187
Years Education				0.078	1.566
Age				0.088	1.745
Model 5b	0.550	0.546	155.369**		
MoCA				0.163	3.446**
Fluency ST				0.652	13.443**

*MoCA* Montreal Cognitive Assessment, *ST* Single Task, *MOS-IS* MOS questionnaire for social survey-Instrumental Support, *GHQ* General Health Questionnaire 12-items, factor 1, *TUG* Timed Up and Go, *LB-IADL* Lawton-Brody Instrumental Activities of Daily Living Scale

**p < 0.01, *p < 0.05

Tracking in the DT condition was significantly predicted by Tracking in the ST condition (Model 1, *R*^2^ adjusted = 0.553, *F* = 159.789, *p* < 0.01), by TUG and CCI (Model 2, *R*^2^ adjusted = 0.090, *F* = 7.343, *p* < 0.01), by MOS-PI (Model 3, *R*^2^ adjusted = 0.029, *F* = 2.091, *p* < 0.05) and by Age (Model 4, *R*^2^ adjusted = 0.148, *F* = 22.006, *p* < 0.01) (see Table 4).

**Table 4:** Multiple regression analysis, with performance in Tracking in Dual Task condition as the dependent variable and cognitive, physical/functional psychosocial and socio-demographic measures as independent variables

Variable entered in model	*R*^2^	*R*^2^ adjusted	*F*	*Β*	*T*
Model 1 (cognitive)	0.556	0.553	159.789**		
MoCA				0.079	1.798
Tracking ST				0.717	16.297**
Model 2 (physical/functional)	0.104	0.090	7.343**		
VREM				0.044	0.710
TUG				− 0.243	− 3.892**
CCI				− 0.133	− 2.205*
LB-IADL				0.076	1.230
Model 3 (psychosocial)	0.056	0.029	2.091*		
MOS-ES				− 0.100	− 1.084
MOS-IS				− 0.104	− 1.319
MOS-PI				0.231	2.041*
MOS-AS				0.030	0.332
GHQ 1				− 0.088	− 1.395
GHQ 2				− 0.200	− 1.870
GHQ 3				0.094	0.861
Model 4 (socio-demographic)	0.148	0.141	22.006**		
Years education				0.105	1.773
Age				− 0.349	− 5.899**

*MoCA* Montreal Cognitive Assessment, *ST* Single task, *VREM* Minnesota Leisure Time Physical Activity Questionnaire, *TUG* Timed Up and Go, *CCI* Charlson Comorbidity Index, *LB-IADL* Lawton-Brody Instrumental Activities of Daily Living Scale, *MOS-ES* MOS questionnaire for social survey-Emotional Support, *MOS-IS* MOS questionnaire for social survey- Instrumental Support, *MOS-PI* MOS questionnaire for social survey-Positive Interaction, *MOS-AS* MOS questionnaire for social survey-Affective Support, *GHQ* General Health Questionnaire 12-items, factors 1, 2 and 3

**p < 0.01, *p < 0.05

Final Model 5a (Table 5) including independent variables that significantly predicted Tracking in DT in the previous analyses produced significant results (*R*^2^ adjusted = 0.590, *F* = 74.374, *p* < 0.01) for Tracking SC, MOS-PI, CCI and Age, which together reached a similar level of significance (Model 5b, *R*^2^ adjusted = 0.591, *F* = 93.323, *p* < 0.01).

**Table 5:** Multiple regression analysis with performance in Tracking in the Dual Task condition as the dependent variable. The statistical significance of the independent variables in Models 1, 2, 3 and 4 (Model 5a) and the statistical significance of the independent variables in Model 5a (Model 5b) are shown

Variable entered in model	*R*^2^	*R*^2^ adjusted	*F*	*β*	*T*
Model 5a	0.597	0.590	74.374**		
Tracking ST				0.699	16.124**
MOS-PI				0.139	3.408**
TUG				− 0.007	− 0.149
CCI				− 0.086	− 2.120*
Age				− 0.124	− 2.642**
Model 5b	0.597	0.591	93.323**		
Tracking ST				0.700	16.373**
MOS-PI				0.140	3.485**
CCI				− 0.086	− 2.122*
Age				− 0.127	− 2.959**

*ST* Single task, *MOS-PI* MOS questionnaire for social survey-positive interaction, *TUG* timed up and go, *CCI* Charlson Comorbidity Index

**p < 0.01; *p < 0.05

## Discussion

The main aim of this study was to determine the variables that influence performance of Fluency and Tracking tasks in the DT condition, to shed some light on the complex relationships between performance of motor and cognitive tasks and different aspects of cognitive, physical/functional, psychosocial and social–demographic aging. The findings showed that only the cognitive variables (MoCA and Fluency in single task) significantly predicted Fluency in the DT condition, but that a more heterogeneous combination of variables influenced Tracking in the DT condition. In the corresponding final model, Tracking performance in a single task was, as expected, the best predictor. However, comorbidity (as a measure of health status), positive interaction (as a marker of social support) and age were also significant predictors. Accordingly, performance in the purely cognitive task (Fluency) in the dual condition was directly influenced by cognitive factors, whereas performance of the cognitive–motor task (which includes tracking, involving following a series of points on a sheet of paper) in the dual condition was more broadly influenced by psychosocial and health factors and age. These results can be interpreted in accordance with those of Pereiro et al. [42], who concluded that the demands involved in hybrid cognitive–motor tasks are better for differentiating levels of disability or frailty.

Comorbidity is often linked to cognitive functions, especially those related to executive processes. Although the underlying mechanisms affecting DT performance are not fully understood, the currently most relevant hypothesis is that the executive demands used for daily living and the executive demands required for selecting and coordinating motor and cognitive tasks share a similar path at the brain level [43]. Social support dimensions measured with MOS were also significant predictors of performance in the dual-task condition, and the specific dimension of Positive Interaction reached significance in the final model (Model 5b), predicting Tracking in the DT condition. Positive social interactions, such as enjoying meeting or doing fun activities with others require selection and coordination of cognitive and motor abilities, which reflect the level of activity in social contexts and appear to be good predictors of performance in dual tasks. This finding is consistent with those reported by Duppen et al. [16] in a review study, i.e., that social participation, neighborhood characteristics and perceived neighborhood experience have a protective role in aging. Nevertheless, according to Duppen et al. [16], the most commonly studied social variables are social network and variables related to social support. Our findings indicate the need to broaden the study of social variables related to the age-related decline, emphasizing those most closely related to social participation, relations with the nearest neighborhood and positive interactions promoting joy and social engagement.

Finally, age emerged as a predictor of Tracking in DT, in combination with health status and positive social interaction. Previous studies found age to be the most influential factor in frailty phenotypes, in combination with other variables such as social resources and the physical facet of the self-report quality of life [44]. In the present study, age was more closely related to the motor component of frailty than to the cognitive aspects.

In daily life, motor and cognitive tasks are performed simultaneously in many situations. Our findings show that degree of physical comorbidity, level of social interaction and age can be used as predictors of DT performance in cognitive–motor tasks. Since we have shown that these tasks will involve higher resource demands in older adults with cognitive impairment and/or some degree of frailty, relationships between cognitive frailty, comorbidity and social participation can be hypothesized. This hypothesis is consistent with broader models of frailty, such as cognitive frailty [17, 45] and social frailty [16, 19]. Some limitations should be pointed. Trials in single and dual conditions are not randomized. Our data collection protocol has been designed following the administration instructions proposed by Della Sala and colleagues [40] who are the authors of the Tracking test. The recommended order is always the same: single task as first trial, and then dual tasks as second. However, this procedure may be conditioning results, are subject to possible learning effects. For other potential predictors of DT performance, such as functional capacity and well-being, larger samples are required to ensure that statistically significant effects will be detected in complex predictive models. Finally, although this work is related to the study of cognitive frailty through its theoretical framework and the selection of relevant variables in our predictive model, this study did not directly quantify the prevalence of cognitive frailty or its relationship with performance in dual tasks. Future studies should make explicit the predictive value of dual tasks for the detection of cognitive frailty as a risk situation for negative health-related outcomes in older adults. Accordingly, future studies should include larger, longitudinal samples and also explore the role of DT performance in detecting and predicting cognitive frailty.

## Data Availability

On demand.
